# Lipid Peroxidation and Antioxidant Protection

**DOI:** 10.3390/biom13091291

**Published:** 2023-08-24

**Authors:** Luca Valgimigli

**Affiliations:** Department of Chemistry “G. Ciamician”, University of Bologna, Via Piero Gobetti 85, 40129 Bologna, Italy; luca.valgimigli@unibo.it; Tel.: +39-0512095683

**Keywords:** autoxidation, peroxyl radicals, hydroperoxyl radicals, kinetics, antioxidants, phenols, catechols, nitroxides, thiols, pyridinols

## Abstract

Lipid peroxidation (LP) is the most important type of oxidative-radical damage in biological systems, owing to its interplay with ferroptosis and to its role in secondary damage to other biomolecules, such as proteins. The chemistry of LP and its biological consequences are reviewed with focus on the kinetics of the various processes, which helps understand the mechanisms and efficacy of antioxidant strategies. The main types of antioxidants are discussed in terms of structure–activity rationalization, with focus on mechanism and kinetics, as well as on their potential role in modulating ferroptosis. Phenols, pyri(mi)dinols, antioxidants based on heavy chalcogens (Se and Te), diarylamines, ascorbate and others are addressed, along with the latest unconventional antioxidant strategies based on the double-sided role of the superoxide/hydroperoxyl radical system.

## 1. Introduction

*Lipid peroxidation* (LP) is a complex phenomenon, first investigated in the early 20th century, consisting in the uptake of molecular oxygen by lipids exposed to air, which was soon recognized as bearing remarkable similarity to hydrocarbon autoxidation [1,2], the formal insertion of one molecule of oxygen in the C-H bond of a hydrocarbon to afford a hydroperoxide: R-H + O_2_ → R-OO-H. Indeed, lipid peroxidation is one embodiment of hydrocarbon autoxidation.

While the direct reaction with ground state (triplet) oxygen is spin restricted and too slow to occur, the transformation of hydrocarbons (or lipids) by oxygen to hydroperoxides and further oxidized products occurs rapidly and efficiently via the intermediation of peroxyl radicals (ROO•), in a chain reaction that can be triggered by a multitude of events in any chemical system, such as in food or in living organisms, and it can be blocked or prevented by *antioxidants* [1,2,3].

Over the last 70 years, extensive research efforts have shown the association of lipid peroxidation with an impressive number of pathological conditions, from arteriosclerosis and cardiovascular diseases to neurological disorders and cancer [1,2,3,4,5,6,7], stemming from the “Free Radical Theory of Aging”, which attributes the progressive decline of functionality associated with aging to the progressive accumulation of damage to biomolecules and essential biological structures, caused by radicals’ overproduction, also referred to as “oxidative stress” [6,7]. Yet the interest in lipid peroxidation has recently seen a boost after the formal recognition in 2012 of ferroptosis as a new form of programmed cell death [8], driven by LP and specifically linked to the pathophysiology of several degenerative diseases [9,10,11]. Even more interesting are pioneering observations that antioxidants can effectively inhibit ferroptosis [12,13] and that either antioxidants or pro-oxidant molecules can be used to modulate it, promising a handle on the connected pathologies [10,13].

Although several valuable reviews have been dedicated to LP and ferroptosis [14,15], the importance of lipid peroxidation is not limited to them. Damage to membrane lipids by radicals and reactive oxygen species (ROS) is perhaps the most important type of oxidative/radical damage in biological systems; not only because of the susceptibility of lipids to radical attack and because it produces the majority of biomarkers commonly used to monitor such damage but because such biomarkers—the LP secondary products—are also major effectors of the damage itself [16], owing to their toxicity and to their ability to alter other biomolecules such as proteins [17], thereby extending the radical/oxidative damage [18].

The aim of this review is to offer a brief up-to-date overview on lipid peroxidation, including the main processes leading to secondary products, with focus on the kinetic aspects regulating the different reactions, so to guide the understanding of the efficacy of the different antioxidant protection strategies. The main type of antioxidants will also be briefly reviewed addressing their efficacy, on kinetic bases, and their mechanism of action.

## 2. The Chemistry of Lipid Peroxidation

### 2.1. The Three Stages of Lipid Peroxidation

**Figure 1:**
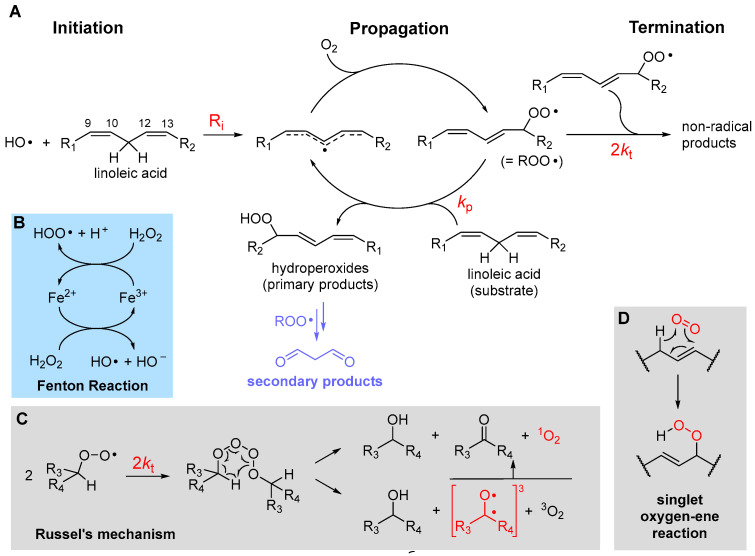
The main steps of lipid peroxidation (**A**), with indication of one main mechanism of initiation (**B**), details of Russel’s mechanism for termination (**C**) and reaction of singlet oxygen with unsaturated lipids (**D**).

LP is a radical chain reaction composed of the canonical three stages of initiation, propagation and termination, summarized in Figure 1 using PUFA as the prototypical oxidizable substrate.

*Initiation*, i.e., the formation of the first lipid-derived radical, can consist in attack by a variety of radical species. In biological systems, these would most commonly be an alkoxyl radical (RO•) or a hydroxyl radical (HO•), e.g., generated by Fenton-type decomposition of hydroperoxides or hydrogen peroxide, catalyzed by transition metals such as iron or copper. Other initiating species could be hydroperoxyl radicals (HOO•), the neutral form of metabolically produced superoxide radical anion (O_2_^•−^), which, however, is not prevailing at pH 7 or higher—the p*K*_a_ is 4.8 in water at 20 °C [19]. Excited triplet states produced by UV irradiation of carbonyl compounds, e.g., in the skin, or radicals produced from water by ionizing radiations would also serve the purpose. Since the attack to lipids occurs typically by formal hydrogen-atom transfer (HAT) from the >CH_2_ in allylic/bis-allylic positions, having a bond dissociation enthalpy (BDE) of about 77/87 kcal/mole, respectively, any radical species X• forming a product X-H with BDE higher than this value would be suitable for the task.

*Propagation* of the oxidative chain occurs by two alternating steps: the reaction of the lipid-derived C-centered radical (>C(•)H; =R•) with oxygen to form the alkylperoxyl radical (ROO•) is extremely fast (rate constant in the range 2–5 × 10^9^ M^−1^s^−1^ [20]), hence the kinetics of propagation is governed by the second step, the reaction of ROO• with a new lipid molecule to afford a new lipid-derived R• radical. Its rate constant *k*_p_ is the most important parameter in evaluating antioxidant strategies (vide infra) and it depends dramatically on the structure of the lipid molecule. As summarized in Table 1, *k*_p_ is negligible for saturated fatty acids (or hydrocarbons) at close to physiologic temperature [21], but it grows by about two orders of magnitude in monounsaturated fatty acids (MUFAs, e.g., methyl oleate) and by about four orders of magnitude with two unsaturations, like in methyl linoleate, which reaches *k*_p_ = 62 M^−1^s^−1^ at 30 °C [22]. This is due to resonance stabilization of the allyl and, particularly, of the bis-allyl radical, resulting by HAT from MUFA and PUFA, respectively. On increasing the degree of unsaturation in fatty acids, the number of >CH_2_ in the bis-allylic position also increases, which explains the almost linear increase in *k*_p_ with the number of double bonds [6], first reported by Porter’s group [23] (Table 1), representing a significant challenge for antioxidant protection (vide infra).

The main products of the overall propagation stage are hydroperoxides (ROOH), the *primary products* of LP [1], and new alkylperoxyl radicals, which are the chain-carrying species in LP. Depending on the lipid structure, formation of other side products like epoxides and endoperoxides might also gain importance (vide infra).

*Termination* of the chain reaction occurs when two radicals quench each other to afford non-radical (diamagnetic) species, which are unable to propagate the chain. Uninhibited LP is carried on by secondary peroxyl radicals, and the best-established termination process is Russel’s mechanism [26], which consists in the formation of a tetroxide that decomposes to afford carbonyl compounds and molecular oxygen (Figure 1). It has lately been clarified that O_2_ released by decomposition of the tetroxide is predominantly in the excited singlet state ^1^O_2_ [27], meaning that oxygen molecules bear all electrons in pairs with antiparallel spin. While the singlet state is the lowest in energy for most molecules, oxygen is the exception and its ground state is a (paramagnetic) triplet biradical, i.e., it has two electrons with parallel spin in (degenerate) antibonding π* orbitals. This, perhaps counterintuitively, makes it much less reactive toward “normal” diamagnetic molecules due to spin restriction, i.e., ^3^O_2_ is much less oxidizing than ^1^O_2_. Other processes have been shown to form singlet oxygen in biological systems. Among them, the reaction of hydrogen peroxide (H_2_O_2_) and lipid hydroperoxides (ROOH) by myeloperoxidase (MPO) in the presence of Cl^−^ ions, which is possibly a defensive mechanism occurring during phagocytosis. It was demonstrated that the process occurs by reaction of H_2_O_2_ or ROOH with HOCl produced by MPO [27]. Earlier work indicated that ^1^O_2_ is quantitatively produced by HOCl reaction on H_2_O_2_ but it is not observed with ROOH [28]. However, subsequent work, addressing the ability of different hydroperoxides to produce ^1^O_2_ upon HOCl reaction, showed that tertiary alkylhydroperoxides are ineffective, while typical lipid-derived secondary ROOH form ^1^O_2_ with an approximately 10% yield via Russel’s mechanism [29]. The decomposition of lipid hydroperoxides in the presence of metal ions or peroxynitrite was also shown to produce ^1^O_2_ with an approximately 10% yield [27]. Decomposition of 7-α-OOH, 6-β-OOH and 5-α-OOH cholesterol hydroperoxides, formed upon cholesterol autoxidation in biomembranes, is also a relevant source of ^1^O_2_ via a Russel’s-type mechanism [27]. Cytochrome c causes the formation of ^1^O_2_ in mitochondria by promoting the autoxidation of cardiolipin [30]. Additionally, singlet oxygen can be produced by photoexcitation of ground state triplet oxygen in the presence of a photosensitizer [6], e.g., in the skin, as summarized in Equation (1). The photosensitizer (PS), such as a carbonyl compound in its ground singlet state, absorbs visible or UV light and is promoted to the excited singlet state, which undergoes intersystem crossing to convert into the excited triplet state. This finally transfers energy to ground state triplet oxygen via a triplet annihilation process, to go back to the ground state converting oxygen into the excited singlet state.

^1^PS + ^3^O_2_ + light → ^1^PS* + ^3^O_2_ → ^3^PS* + ^3^O_2_ → ^1^PS + ^1^O_2_ (1)

Singlet oxygen is unable to restart the autoxidation chain; however, it directly reacts with unsaturated lipids via an oxygen-ene reaction to afford the lipid hydroperoxides (Figure 1) [6].

### 2.2. The Rate of Lipid Peroxidation

Chain termination typically quenches two peroxyl radicals in a fast radical–radical reaction with rate constant 2*k*_t_. Although 2*k*_t_ is much higher than the rate constant *k*_p_ for propagation (see Table 1), the actual rate of termination also depends on the square of the steady-state concentration of the chain-carrying peroxyl radical, which is very low, typically in the micromolar-to-nanomolar range. Therefore, it can be estimated that, following an initiation event, >100 propagation cycles occur before termination takes place in LP of unsaturated lipids, implying that one single initiating radical can damage hundreds of lipid molecules, which explains why antioxidants are necessary. 

In the absence of antioxidants, the rate of peroxidation of a lipid substrate RH, at a constant rate of chain initiation *R*_i_, follows Equation (2) [21,22,24,31], i.e., it is dictated by the ratio of the rate constant for propagation over the square root of the rate constant for termination. The kinetic quantity *k*_p_/(2*k*_t_)^1/2^ is named the “oxidizability” of the specific lipid substrate; it increases with unsaturation (Table 1) and it determines how important antioxidant protection is for the specific substrate.

(2) $$-\frac{d\left[O_{2}\right]}{dt}=-\frac{d\left[RH\right]}{dt}=\frac{d\left[ROOH\right]}{dt}=\frac{k_{p}}{\sqrt{{2k}_{t}}}\times \sqrt{R_{i}}\times \left[RH\right]+R_{i}$$

### 2.3. Light as a Product of Lipid Peroxidation

The termination stage of LP produces high-energy (transient) species that can deactivate to ground state or lose excess energy by emitting light, which can therefore be regarded as a product of LP. One important example is singlet oxygen, ^1^O_2_, which can return to a ground state triplet by giving weak phosphorescence in the near infrared region at 1270 nm [27]. This chemoluminescence (CL) phenomenon has been extensively exploited to probe the formation of ^1^O_2_ during LP [27,28,29] and strategies have been developed to enhance CL in biological systems to improve detection sensitivity [32].

Emission of chemoluminescensce in the visible region is also observed during LP and it is attributed to excited carbonyls in the triplet state, such as those formed in Russel’s termination mechanism (Figure 1C), although singlet oxygen, e.g., formed in the same process, also contributes [33]. It has also been proposed that part of the excited triplet carbonyls affording CL are formed by an alternative termination mechanism consisting in cyclization of the alkylperoxyl radical to a dioxyethane followed by its thermolysis [34]. Emission can be enhanced by additives like 9,10-dibromoanthracene and it is proportional to the rate of LP [33]. Interestingly, the time-based monitoring of CL has been developed as a method to monitor the kinetics of LP both in animal and vegetable lipids [33,35], which has also been fruitfully applied to study the activity of antioxidants [36,37]

### 2.4. Further Insights into Chain-Propagation Reactions

#### 2.4.1. β-Fragmentation of the Peroxyl Radical

**Figure 2:**
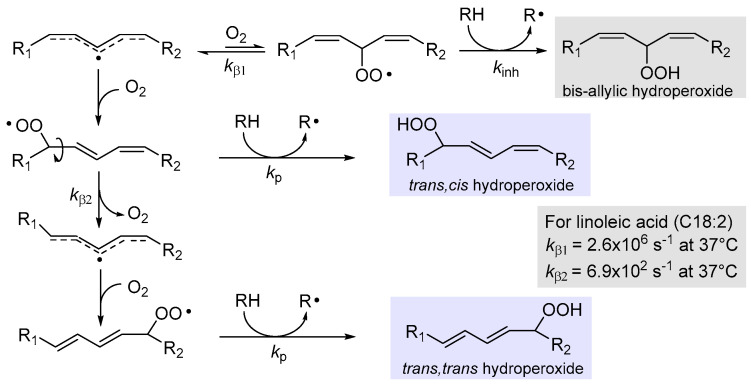
Schematic representation of β-fragmentation reactions in peroxyl radicals competing with H-atom abstraction from a donor RH, which is a lipid molecule (*k*_p_) or an antioxidant (*k*_inh_).

Addition of oxygen to lipid radical R• to afford the peroxyl radical ROO• is a reversible process and its back reaction, named β-fragmentation, has a rate constant *k*_β_, which depends on the stability of the (reformed) carbon-centered radical (Figure 2). While it is generally too slow compared to the forward reaction to affect the rate of chain propagation, under some conditions it can compete with the subsequent propagation step, the reaction of ROO• with the lipid molecule RH to afford the hydroperoxide primary product ROOH [38]. Therefore, it determines the distribution of isomeric hydroperoxide products, since *cis,trans* hydroperoxides are formed when propagation outcompetes β-fragmentation, hence they are the kinetic products of LP, while *trans,trans* hydroperoxides are the thermodynamic products, forming when β-fragmentation allows isomerization to take place [23,38]. 

This reaction was thoroughly investigated in Porter’s group who found that, upon calibration of the rate of β-fragmentations, these unimolecular reactions could be used as a “radical clock” to measure the kinetics of competing H-abstractions, by the analysis of the hydroperoxide product ratio from autoxidation [39]. For instance, they found that the rate constant for fragmentation of *cis,trans* hydroperoxides from linoleic acid (Figure 2) was *k*_β2_ = 6.9 × 10^2^ s^−1^ (at 37 °C) and would be useful to measure rate constants for propagation (*k*_p_). The fragmentation of bis-allyl hydroperoxide is instead much faster (*k*_β1_ = 2.6 × 10^6^ s^−1^ at 37 °C) and the corresponding hydroperoxide would never be formed in the absence of a much faster H-atom donor such as α-tocopherol (α-TOH) or other antioxidants; therefore, they used this radical clock to measure the rate constant of inhibition *k*_inh_ for antioxidants (vide infra) [39].

#### 2.4.2. Hydrogen-Atom Abstraction vs. Radical Addition: Formation of Primary Epoxides

**Figure 3:**
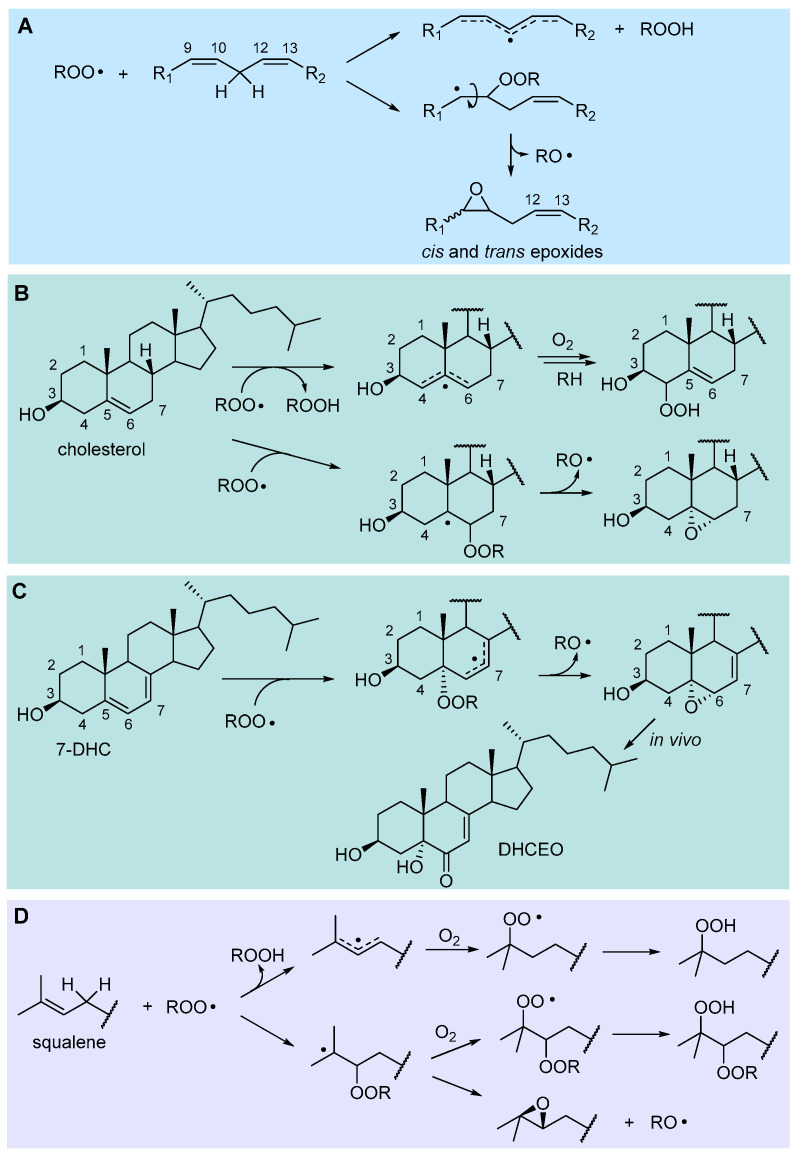
Formation of epoxides during the peroxidation of PUFA (**A**), of cholesterol (**B**), of 7-dehydrocholesterol (**C**) and of squalene (**D**).

Although chain propagation in PUFAs such as linoleic acid is carried out mainly by H-atom abstraction from the bis-allylic position to afford the stabilized pentadienyl-type radical, addition of the ROO• radical to one of the C=C double bonds can also compete, to some extent, being favored by the contribution of polar effects in the transition state [40]. The resulting alkylperoxyalkyl radical would rapidly undergo a homolytic substitution (S_H_^i^) reaction, forming an epoxide and releasing an alkoxyl radical RO• (Figure 3), which would rapidly attack another lipid molecule, propagating the chain. Therefore, from a kinetic perspective, H-abstraction and addition are difficult to distinguish, besides the fact they afford different primary oxidation products, hydroperoxides or epoxides, respectively. 

The relative importance of addition might depend on the experimental conditions and would certainly depend on the lipid structure. Confirmation of its relevance comes from the identification of both 9,10- and 12,13-epoxides among the oxidation products of methyl linoleate [41], while epoxyeicosatrienoic acids formed via peroxyl radical addition were found in the peroxidation of arachidonic acid (C20:4) [42]. 

In the autoxidation of cholesterol, epoxides at C5,6 (α+β) were found to account for about 12% of the total oxidation products [43], demonstrating the importance of addition in chain propagation. A detailed study by Pratt’s group showed that addition at C6 had similar activation energy (∆G^‡^ = 17.6 kcal/mol) to H-abstraction at C7-H (BDE = 83.2 kcal/mol, ∆G^‡^ = 17.5 kcal/mol), being faster than H-abstraction at C4-H (BDE = 89.0 kcal/mol, ∆G^‡^ = 19.4 kcal/mol) which would afford, respectively, the 7-OOH and 4-OOH hydroperoxides. Instead, the 6-OOR resulting from addition would undergo very rapid (∆G^‡^ = 7.3 kcal/mol) transformation to the 5,6-epoxide [43], as shown in Figure 3.

7-Dehydrocholesterol (7-HDC) is formed as the last step before cholesterol in the biosynthesis path that starts from squalene, and its metabolism is implicated in Smith−Lemli−Opitz syndrome (SLOS), a devastating autosomal disorder with a range of phenotypical expressions, including malformations and neurological deficits [38]. SLOS is characterized by much-elevated levels of 7-HDC (and low levels of cholesterol) due to defects in the gene encoding for 7-dehydrocholesterol reductase. 7-HDC is one the most oxidizable lipids in biological systems with a *k*_p_ 200-fold higher than cholesterol and 10- to 40-fold higher than PUFA (Table 1), which reacts with peroxyl radicals mainly H-abstraction, at C9-H or by addition at C5 (at variance with cholesterol) to afford a stabilized allylic radical [38]. This yields the 5α,6α-epoxide (Figure 3) that, in vivo, is converted to 3β,5α-dihydroxycholest-7-en-6-one (DHCEO), which has been used as a biomarker in SLOS [38,43].

The autoxidation of squalene, the polyunsaturated triterpenic precursor of cholesterol and phytosterols, has recently been kinetically characterized in our group, finding a *k*_p_ of 68 M^−1^s^−1^, just slightly higher than that of methyl linoleate and 6-fold that of cholesterol (Table 1), which is due mainly to H-abstraction at repeating allylic positions, with significant contribution of ROO• addition, to afford a tertiary alkyl radical developing into an epoxide, as illustrated in Figure 3 [24].

A peroxyl radical clock designed for measuring both H-atom abstraction and radical addition has recently been described and its application to a variety of unsaturated hydrocarbons indicates that addition grows in importance in conjugated dienes and polyenes; these include retinol (vitamin A) and carotenoids, conjugated linoleic acid (CLA 18:2) and conjugated linolenic acid (CLA 18:3) [44].

#### 2.4.3. Release of HOO• and Chain-Transfer Processes

**Figure 4:**
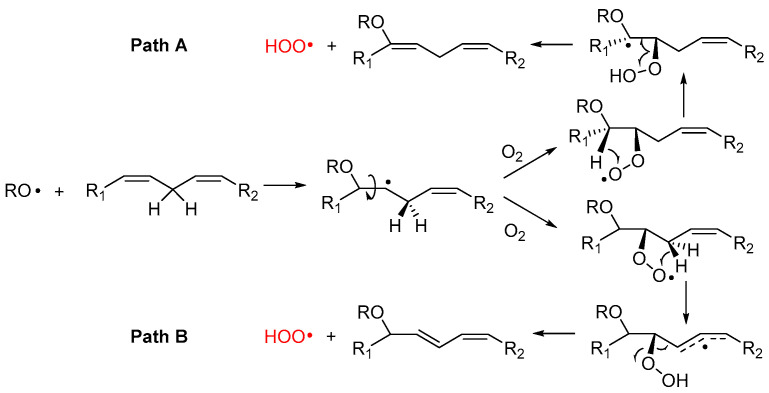
Mechanistic proposal for the release of HOO• during the autoxidation of methyl linoleate.

Alkoxyl radicals released via S_H_^i^ reaction upon epoxide formation have very high reactivity toward unsaturated lipids and, besides H-atom abstraction, they undergo very fast addition to C=C double bonds [45,46], forming a β-alkoxyalkyl radical that rapidly reacts with oxygen (Figure 4). The resulting β-alkoxyalkylperoxyl can undergo intramolecular H-atom abstraction (1,4-HAT) either in Cα to the RO- group, to form a radical stabilized by resonance with the oxygen lone pair (Path A), or from the allylic >CH_2_ to form a radical stabilized by resonance with the double bond (Path B). In both cases, the resulting hydroperoxide can undergo fragmentation to release the hydroperoxyl radical HOO• and reform the C=C double bond in the lipid structure. The release of HOO• has important consequences in the antioxidant protection of lipids (vide infra).

Although this process has never been demonstrated to occur in lipids like PUFA, similar reactions are known for simpler unsaturated hydrocarbons. The best established is 1,4-cyclohexadiene, which upon attack by alkylperoxyl radicals releases HOO• and converts to benzene [47], and the monoterpene γ-terpinene, which undergoes identical chemistry affording HOO• and *p*-cymene [48]. In those hydrocarbons, the two steps, 1,4-HAT and elimination, are concerted and occur in a single transition state with a low barrier [49]. Pratt and coworkers were able to demonstrate that the reaction also occurs for simple monounsaturated hydrocarbons (like cyclooctene), albeit it does so stepwise, as depicted in Path A of Figure 4, and its kinetics is favored by quantum tunneling in the 1,4-HAT [49].

We have recently suggested the same mechanism to account for some non-classical behavior in the inhibited autoxidation of squalene [24], and ongoing studies in our group indicate a similar chemistry would take place during the autoxidation of methyl linoleate in micelles. In the case of PUFA, however, the possibility to form conjugated double bonds in the product suggests that Path B could also be a viable mechanism.

The release of HOO• has kinetic consequences, as these radicals can propagate the oxidative chain in bulk lipids—i.e., it represents a chain-transfer process—but, owing to their hydrosolubility, they could export the unpaired electron in the aqueous phase in heterogenous systems. As will be discussed in Section 5, it also has major importance for the antioxidant protection of lipids, under some conditions.

#### 2.4.4. Formation of Endoperoxides

Product distribution can become quite complex in highly unsaturated lipids as it can involve intramolecular radical addition to double bonds to yield cyclized products.

**Figure 5:**
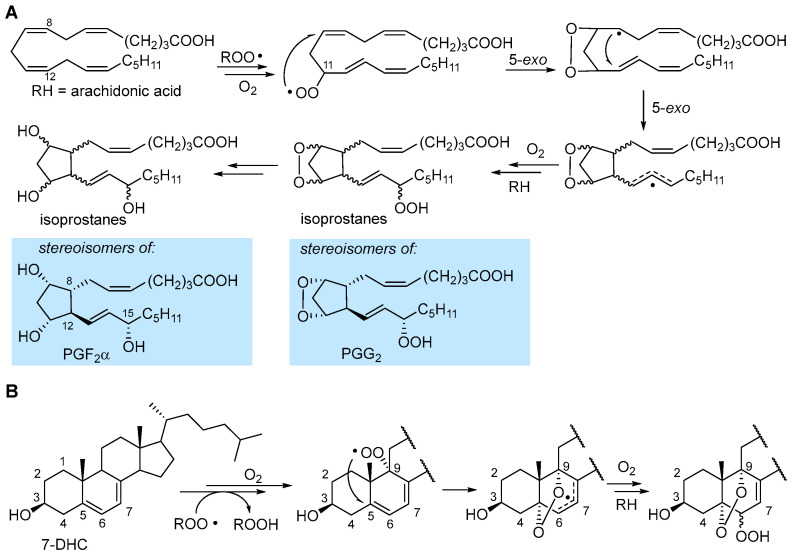
Formation of endoperoxides during the propagation of lipid peroxidation of arachidonic acid (**A**) and 7-dehydrocholesterol (**B**).

In the autoxidation of arachidonic acid in the absence of fast H-atom donors (e.g., α-TOH), the peroxyl radical can undergo 5-*exo* cyclization occurring with a monomolecular rate constant of about 800 s^−1^ which outcompetes β-fragmentation with *k*_β_ of about 140 s^−1^ [1]. The result is the formation of an endoperoxide still bearing a C-centered radical (Figure 5), which undergoes another 5-*exo* cyclization, to form another alkyl radical, and further autoxidation steps to ultimately yield endoperoxide products or intermediates, which are stereoisomeric to some of the prostaglandins (e.g., PGG_2_ and PGF_2_α) produced by cyclooxygenase (COX) enzyme [31], therefore generally named isoprostanes.

In 7-HDC, autoxidation proceeds both by ROO• addition to yield the epoxide (Figure 3) and by H-atom abstraction preferably from C9-H [31,38]. The resulting peroxyl radical was found to undergo 5-exo cyclization at C5 to yield a stabilized allyl radical that continues the autoxidation to afford a mixture of α-5,9-endoperoxides-α&β-6-hydroperoxide [38], as shown in Figure 5. In general, endoperoxide formation is observed when the unsaturated lipid geometry allows for 5-*exo* cyclization, possibly yielding an unstrained or resonance-stabilized radical.

### 2.5. Peroxidation of Intact Triglycerides and Phospholipids

Most of the knowledge and understanding of the chemistry and related kinetics of lipid peroxidation have been developed using isolated PUFAs and MUFAs and their simple monoesters (e.g., methyl linoleate) as model compounds, along with biologically relevant yet specific compounds, like cholesterol and some of its congeners, or it has been mutuated from knowledge on simpler hydrocarbons. However, on quantitative grounds, the majority of lipids in biological systems are phospholipids (e.g., in cell membranes) and triglycerides (e.g., in adipocytes and liporoteins). Unfortunately, only a limited number of studies have specifically addressed their peroxidation chemistry, owing to their complexity, showing, however, some distinctive features.

**Figure 6:**
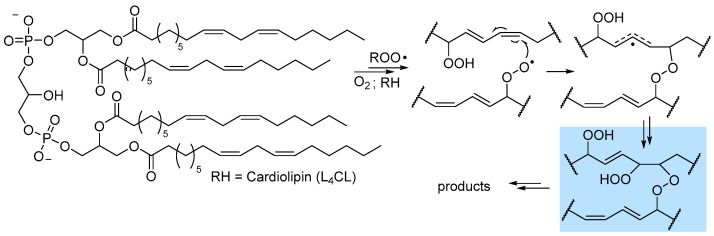
Formation of arm-to-arm peroxides in the peroxidation of mitochondrial cardiolipin.

A pioneering study by Antunes et al. investigated the kinetics of peroxidation of phosphatidilcholine multilamellar liposomes in buffered water. They studied the liposomes of both 1-palmitoyl-2-linoleoyl-sn-glycero-3-phosphocholine (PLPC) and 1,2-dilinoleoyl-sn-glycero-3-phosphocholine (DLPC), containing, respectively, one and two oxidizable linoleic acid residues per phospholipid molecule [25]. For PLPC, where only one chain is really oxidizable, they found k_p_ = 16.6 M^−1^s^−1^ and 2k_t_ = 1.27 × 10^5^ M^−1^s^−1^ at 37 °C, hence they were both significantly lower that those recorded for methyl linoleate in organic solution (Table 1). Interestingly, with DLPC, carrying two oxidizable chains per molecule, they were able to distinguish two rate constants k_p_ of 13.6 M^−1^s^−1^ and of 5.1 s^−1^ for intermolacular and intramolecular chain propagation, respectively, while chain termination gave the rate constant 2k_t_ = 1.02 × 10^5^ M^−1^s^−1^ at 37 °C [25]. This clearly shows that the presence of two oxidizable chains in the same lipid molecules changes the kinetic behavior. Stemming from their results, Porter’s group investigated, by the radical clock method, the kinetics of chain propagation for PC liposomes always containing palmitic acid esterified in position 1 of the gycerol and one PUFA in position 2: linoleic acid (C18:2; PLPC), used as reference, arachidonic acid (C20:4; PAPC), eicosapentaenoic acid (C20:5; PEPC) and docosahexanoic acid (C22:6; PDPC) [23]. The measured k_p_ values were proportional to the number of double bonds, as also seen for isolated PUFA in organic solution, but the absolute values were much lower (see Table 1), i.e., 35, 115, 145 and 172 n^−1^s^−1^, respectively, referring to the mole fraction of the oxidizable lipid in the lipsomes, instead of the molar concentration in solution [23]. Interestingly, they found a different kinetic behavior of the peroxyl radicals in C9 or in C13 in linoleoyl residue, indicating that oxidation in liposomes depends on the position of the formed peroxyl radical. While the above results refer to lipids having only one oxidizable PUFA residue, subsequent studies on 1,2-dilinoleoyl-glycero-3-phosphocholine (DLPC) and on tetralinoleoyl cardiolipin (L_4_CL), a mitochondria-specific phospholipid carrying four linoleic acid residues, proved that arm-to-arm attack by peroxyl radical is relevant and it can form interarm peroxide (-O-O-) bridges by addition to double bonds (Figure 6), with consequences on the formation of toxic electrophilic fragmentation products like 4-hydroxynonenal (vide infra) [50].

The kinetics of peroxidation of triglycerides is more complex than that of the isolated fatty acids in solution, owing to arm-to-arm propagation processes, which are more relevant in dilute solution than at high concentration or in the bulk [51]. To date, the only complete kinetic characterization of an intact natural triglyceride is that of sunflower seed oil (SSO) recently reported by our group [24]. Each trigyceride molecule statistically contains 1.7 chains of linoleic acid and its oxidizability was found to be roughly 1.7-fold that of linoleic acid; however, to our surprise, the rate constant k_p_ was just slightly higher than that of linoleic acid (67 vs. 62 M^−1^s^−1^), hence the higher oxidizability is due to slower termination (see Table 1), possibly owing to steric impairment in Russel’s mechanism [24].

Clearly, more data would be desirable for complex lipids like phospholipids and triglycerides, particularly in the light of their importance for the onsetting of antioxidant strategies in biological systems.

## 3. Secondary and Late Products of Lipid Peroxidation

Following the formation of primary oxidation products of LP, namely primary hydroperoxides and primary epoxides, formed directly in chain propagation, their further oxidation, followed or accompanied by other reactions, yields a multitude of secondary products. Several such products are electrophiles and can undergo other reactions, e.g., with proteins, and they have cell signaling functions. Hence, they are toxic or might have some active biological role and have been regarded as biomarkers of oxidative damage, prompting great attention and major efforts in the development of analytical techniques to detect them [16,52,53,54,55,56,57,58,59,60,61].

### 3.1. Formation of Electrophilic Carbonyl Compounds

**Figure 7:**
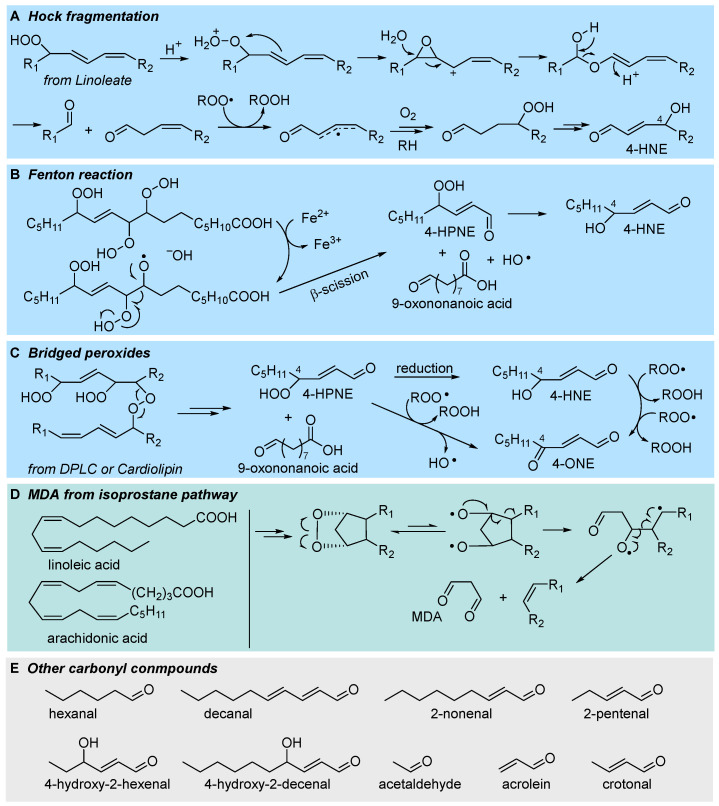
Formation of electrophiles as late products of LP: (**A**) 4-hydroxynonenal (4-HNE) from Hock rearrangment and fragmentation; (**B**) 4-HNE from Fenton reaction on dihydroperoxides; (**C**) 4-HNE and 4-oxononenal (4-ONE) from bridged peroxides; (**D**) malondialdehyde (MDA) from isoprostane pathway; (**E**) other common carbonyl compounds.

The most regarded secondary product of LP, which is also among the most relevant LP biomarkers in biological systems, is 4-hydroxynonenal (4-HNE), formed as a late product of oxidation of linoleic acid [52,55,60]. It is an α-β unsaturated aldehyde with very high reactivity towards biological nucleophiles (e.g., amino acid residues and DNA bases), which grants both its role as a signaling molecule and its toxicity. Despite its importance, 4-HNE is not unique but a member of a broad family of short-chain aldehydes and ketones, often bearing conjugated unsaturation in the hydrocarbon chain, all characterized by the marked electrophilic character and all deriving from fragmentation of the hydrocarbon backbone of oxidized lipids; some of these carbonyl compounds are collected in Figure 7. Among them, two arguably stand out: 4-oxononenal (4-ONE), the oxidized form of 4-HNE, being orders of magnitude more reactive as an electrophile [6,58], and malondialdehyde (MDA), a dicarbonyl compound that is arguably the best-known marker of LP, owing to its facile detection (by spectrophotometry or HPLC) upon derivatization with thiobarbituric acid (the so-called TBARS assay) [52]. The mechanism by which 4-HNE forms from oxidized lipids has been highly debated, and several hypotheses have been proposed, which are not mutually exclusive and, possibly, might all be relevant to some extent. 

The most classical mechanism is perhaps the one based on Hock rearrangement and fragmentation of the primary hydroperoxide in the presence of an acid catalyst [6,31]. The formed carbonyl compounds can then undergo further autoxidation to afford 4-HNE, as depicted in Figure 7A. Another well-established mechanism is based on Fenton-type cleavage of one hyroperoxyl group in a multiply oxidized linoleyl residue to afford an alkoxyl radical that undergoes fragmentation [6,55]. The reduction of the formed 4-hydroperoxynonenal affords 4-HNE (Figure 7B).

Perhaps the most relevant mechanistic proposal, by Porter’s group, stems from the bridged peroxides formed by arm-to-arm propagation in LP of phospholipids, which we have discussed in Section 2.3. Breaking of the peroxide bridge affords 4-HNE and 4-ONE (as summarized in Figure 7C), respectively, by reduction of the -OOH group and by formal water elimination (actually H-abstraction from Cα and elimination of •OH radical from -OOH) [50]. Even more controversial is the mechanism of formation of MDA, which could follow different routes, including processes not necessarily involving LP [62]. One significant mechanism is along the pathway to isoprostanes (see Figure 5), actually in competition with their formation, which relies on the spontaneous or induced breaking of the endoperoxide [31], as illustrated in Figure 7D. Although the formation of endoperoxides via 5-*exo* peroxyl addition and 5-*exo* alkyl addition is normally described for arachidonic acid’s LP [31,62], actually only two non-conjugated double bonds are necessary to form the dioxabicyclo[2.2.1]heptane structure, hence this mechanism can also apply to linoleic acid or other PUFAs.

Carbonyl compounds are also formed as secondary products of cholesterol hydroperoxides; for instance, Hock fragmentation and further oxidation of cholesterol 5-hydroperoxide yield secosterols [31], which have been implicated in several diseases [63,64,65].

### 3.2. Formation of Isoprostanes

Formation of isoprostanes, from sequential steps following the formation of the peroxyl radical in the peroxidation of arachidonic acid, is described in Section 2.3 and illustrated in Figure 5. Isoprostanes are stereoisomers of some of the otherwise identical prostaglandins, e.g., PGH_2_, PGG_2_ and PGF_2_α, formed (instead) with full stereochemical control by cyclooxygenase (COX 1 and 2 in humans), still from arachidonic acid, released by a phospholipase during the inflammatory cascade [38]. Owing to their high specificity, isoprostanes have gained importance as the most reliable markers of LP and oxidative stress in biological systems, particularly in relation to some conditions which they directly affect [4,64,65,66,67,68]. Among them, 8-epi-PGF2α (also called 8-isoprostane, 8-isoPGF2α, iPF2α-III and 15-F2t-IsoP), the epimer in C8 of PGF_2_α, is the best-established marker of oxidative stress in human studies, owing to its stability and selective urinary excretion, which makes its monitoring sensitive and conveneint [4,67]. The autoxidation of arachidonic acid occurs with preferential formation of 8-, 9-, 11- or 12-peroxy radicals; each of them generates one class of F2-IsoP [67,68] and each of the four classes consists of 16 stereoisomers, since the autoxidation and 5-exo ring closures proceed with no stereochemical control (Figure 5), resulting in a total of 64 F2-IsoPs that can be formed during peroxidation of arachidonic acid [67].

### 3.3. Interaction of LP Products with Amino Acids and Proteins

**Figure 8:**
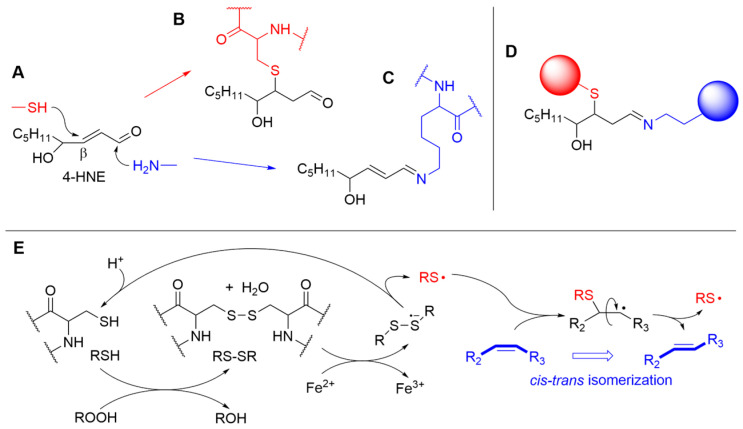
Examples of lipid–peptide interaction in peroxidation: (**A**) favorite nucleophilic attack sites in 4-HNE; (**B**) resulting Cys-HNE adduct; (**C**) Lys-HNE adduct; (**D**) protein cross-linking by 4-HNE; (**E**) lipid cis–trans isomerization caused by the reversible addition of Cys-derived thiyl radical.

Carbonyl secondary products show high reactivity with cell nucleophiles, namely DNA and proteins [6,58]. The reaction with proteins is particularly noteworthy as it interferes with their biological functions in both ways and can express either cytoprotective or damaging roles [6,18]. The most important nucleophiles in peptides are thiols (e.g., the -SH of cycteine residues) and amines (e.g., the side chain -NH_2_ of lysine, the inidazolyl residue in histidine, the guanidine residue in arginine) [69]. Taking conjugated alkenals like 4-HNE as a model, amino groups preferably undergo Schiff-base formation with the carbonyl, while other nucleophiles preferably undertake 1,4-Michael-type addition to the C=C; the subsequent reaction of 4-HNE with both an amine residue and a thiol residue of different proteins can result in protein cross-linking [6,58], as exemplified in Figure 8. Second-order rate constants for the reaction of 4-HNE and 4-ONE with model amino acid derivatives shows that 4-ONE is more reactive by two orders of magnitude with thiols (e.g., reaction with GSH at 23 °C, pH 7.4: k_2_ is 1.33 and 150 M^−1^s^−1^, for 4-HNE and 4-ONE, respectively), while it is only about one order of magnude more reactive with other nucleophiles (e.g., reaction with N-acetylhistamine at 23 °C, pH 7.4: k_2_ is 2.1 × 10^−3^ and 2.2 × 10^−2^ M^−1^s^−1^, for 4-HNE and 4-ONE. respectively) [69]. Protein alkylation is the main process responsible for the biological activity of LP secondary products (vide infra).

Interestingly, in parallel to the influence of lipid oxidation products in the structure and functions of proteins, oxidative damage to peptides and proteins, particularly concerning cysteine and methionine residues, would cause radical damage to lipids, altering their stucture and functions [70,71,72,73].

A main mechanism for peptide-to-lipid damage transfer was extensively investigated in Chatgilialoglu-Ferreri’s group and consists of cis–trans isomerization of C=C double bonds in MUFA and PUFA, caused by reversible addition of thiyl radicals (RS•) and sulfhydryl radicals (HS•/S^−•^) generated from cystein, as exemplified in Figure 8E. Thiyl radicals undergo fast addition to cis C=C double bonds with a bimolecular rate constant of 1.6 × 10^5^ M^−1^s^−1^ (for methyl oleate), but the adduct undergoes rapid fragmentation, which yields the trans isomer approximately 10-fold faster than the cis isomer (viz. 1.7 × 10^7^ s^−1^ vs. 1.6 × 10^8^ s^−1^ for methyl oleate), thereby converting the less stable cis isomer of the lipids into the stable trans isomer [71]. Although formally this process does not belong to LP, as the lipids have not changed their oxidation state, isomerization, occurring, for instance, in cell membrane phospholipids, alters membrane function similarly to LP (vide infra) by affecting its fluidity; additionally, the two processes are interconnected, and both are involved in the development and control of ferroptosis [74].

## 4. Biological Consequences of Lipid Peroxidation

Lipid peroxidation, oxidative damage to other essential biomolecules, like proteins and DNA, and the generation in biological systems of oxidizing radicals not adequately balanced by antioxidant defenses have long been associated with different degenerative pathologies, particularly with cardiovascular diseases and cancer [1,2,3], and have been referred to as oxidative stress (OS), although the actual causal interplay has long escaped full rationalization. Understanding has enormously progressed in recent years and a number of valuable reviews have addressed it [4,6,10,14,15,18,57]. Therefore, we will limit this section to a brief overview, aimed at highlighting the potential of antioxidant strategies, presented in Section 5.

It is now recognized that LP is necessary to cell physiology, to maintain redox homeostasis, and it has essential signaling functions and protective roles. However, dysregulation causes alteration of cell metabolism and/or cell death and it is involved in a multitude of pathologic conditions.

### 4.1. LP and Membrane Integrity and Functions

Modification of cell membranes is the most direct biological consequence of LP. Accumulation of hydroperoxides in the phospholipid bilayer alters their physical–chemical character by increasing the polarity of phospholipids and by altering their aggregation and hence membrane structure. Studies on giant unilamellar vesicles (GUVs) as model systems showed that accumulation of hydroperoxides causes an increase in the area which is proportional to the extent of peroxidation, and it is accompanied by a decrease in thickness of the membrane. Additionally, changes in the liquid–gel transition temperature and phase separations in the lipid domain were observed [75]. Oxidation of cholesterol extends those changes but, alone, is insufficient to cause them. On extending the oxidation and releasing small-chain secondary products, permeability increases, and the integrity is compromised with creation of pores [75]. The mechanical properties of the membrane are significantly altered, with the stretching module decreasing linearly with the conversion into peroxidized lipids, from 200 mN m^−1^ for native lipids to 50 mN m^−1^ for fully oxidized bilayers [76]. Mechanical changes and increases in the surface area were judged to be the consequence of changes in phospholipid chain conformation, caused by the introduction of polar -OOH groups, with increased probability to settle at the lipid–water interface [75]. When changes to membrane structure are sufficiently extended to cause pores or altered permeability, the unavoidable consequence is cell death, which is one of the main effector strategies of ferroptosis (vide infra).

### 4.2. LP and Cell Signaling

ROS and LP electrophiles (LPEs) are two main families of players in non-enzymatic cell signaling; among them, LPEs have a prominent role due to generally higher persistency and ability to diffuse from the immediate surroundings of the generation site [57]. Their main signaling mechanism is by forming protein adducts. Due to the different location of the reacting amino acid residue and to the protein to which the amino acid pertains, this would result in modifying cell metabolism in different ways, both by activating and deactivating specific pathways [18]. At low levels (0.1–1 µM), 4-HNE produces adducts that promote the biosynthesis of antioxidant and detoxifying enzymes, thereby having a protective role. The main mechanism is the reaction with Cys residues of cytoplasmic Keap1 protein, which is part of the nuclear factor erythroid 2-related factor 2 (Nrf2) signaling system. A dimer of Keap1 protein normally holds the transcription factor Nrf2 in the cytoplasm, inhibiting its function. Upon alkylation by 4-HNE, a change in Keap1 conformation causes the release of Nrf2, which translocates into the nucleus and binds to Maf small protein. The resulting heterodimer binds DNA at antioxidant-responsive element (ARE) sites to upregulate genes coding for antioxidant enzymes, such as thioredoxin reductase, and for proteins that cause increased glutathione (GSH) levels [6,18]. However, at higher doses (50 µM), 4-HNE also creates adducts with histones, modifying protein expression at the DNA level, e.g., it blocks histone H2A acetylation, thereby impairing gene expression and contributing to the vulnerability of DNA to apoptosis. 4-HNE also affects the activity of NFκB transcription factor in opposite directions depending on the dose, with resulting anti-inflammatory of proinflammatory effects. The formation of MDA–protein adducts is associated with a proinflammatory reaction throughout the entire organism, via the activation of Th17 lymphocytes, which triggers autoimmune reactions [77]. Additionally, MDA leads to collagen cross-linking causing a loss of elasticity and disturbance in tissue remodeling, with systemic consequences, particularly on the blood vessel system [78]. The above are just examples of the complex pattern of influence of the metabolic machinery elicited by LPEs’ signaling role.

Lipid hydroperoxides themselves are signaling factors and are key players in inflammatory processes. Besides being the primary products of LP formed in the propagation stage, they are also formed by direct oxidation of lipids by singlet oxygen (see: Section 2.1) as a form of defense from such highly reactive species and by enzymatic processes, most notably by lipoxygenases (LOXs), a family of dioxygenases converting lipids to their hydroperoxides that can further transform into other hormone-like signaling molecules (e.g., tromboxanes, leucotrienes, lipoxines and resolvines), which modulate the activity of the innate immune system (e.g., the action of macrophages) in both ways, exerting proinflammatory or anti-inflammatory roles [79]. Also, untransformed LOX-generated ROOHs primarily exert a signaling role towards the immune systems (typically proinflammatory), they directly alter membrane structure and permeability (see: Section 4.1) and might indirectly modify cell gene expression by changing the overall cell redox state [79]. LOX-generated hydroperoxides have also been attributed a key role in regulation of ferroptosis (see: Section 4.5) [80].

### 4.3. LP Association with Cancer and Apoptosis

Association between different markers of oxidative stress and cancer has been extensively reported in recent decades. Metabolic requirements of cancer cells are higher due to their rapid proliferation, which makes mitochondrial ROS generation higher than in non-transformed cells. Redox status and ROS are also of particular importance in tumor cell signaling associated with cancer progression. Therefore, compared to healthy non-malignant cells, cancer cells have altered redox homeostasis and higher levels of LP; in many types of tumor cells, these higher levels support their growth, proliferation and survival [81]. Therefore, higher levels of LP products and biomarkers are expected in association with cancer.

For instance, in a large population-based cohort study with 14 years of follow-up, it was found that a positive association exists between 8-isoprostane urinary levels and occurrence of lung cancer but not of other cancers. Actually, in smokers 8-isoprostane levels were inversely correlated with occurrence of prostate cancer, indicating a protective effect, possibly because higher OS levels associated with smoking, combined with testosterone, activate apoptosis, thereby protecting from cancer development in testosterone-sensitive tissues [68]. A recent study on the relationship between 4-HNE and prostate cancer based on metabolic profiling using LC-ESI-QTOF-MS and GC-EI-Q-MS revealed increased 4-HNE–protein adduct levels in the plasma of cancer patients while there were no 4-HNE–protein adducts in prostate carcinoma tissue [60]. Interestingly, while higher levels of LPEs are typically associated with cancer, some of them also promote defensive mechanisms.

A main defense mechanism against cancer development is apoptosis, a physiological mechanism of programmed cell death that can be triggered both by *death receptor* activation and by metabolic changes in the cell [64]. 4-HNE can modify the structure of the MDM2 protein, breaking down the MDM2–p53 complex. As a consequence, the p53 factor is activated and translocates to the nucleus, where it promotes the transcription of proapoptotic proteins, such as Bax and caspase-3 [66]. 4-HNE may also interact with other proteins; for example, with AKT kinase, which has inhibiting roles, thereby leading to a significant increase in apoptosis. 4-HNE and 4-hydroxyhexenal further promote apoptosis by other mechanisms, which have recently been reviewed [66].

### 4.4. LP and Neurological Disorders

The number of deaths associated with neurological disorders including neurodegenerative diseases like amyotrophic lateral sclerosis and Parkinson’s, Alzheimer’s and Huntington’s diseases, as well as other diseases that involve neurodegenerative processes, like diabetes, have increased worldwide with time [82]. Additionally, these diseases have an important social impact as they compromise patients’ quality of life. Although the causes of neurodegeneration are often unclear, OS and LP, along with inflammation, are recognized as prevailing molecular pathways leading to these diseases [82]. Some known inducers of neurodegeneration like arabin and methylmercury have been found to cause it through induction of LP and reduction of antioxidant defenses, or at least in parallel to inducing them [82,83], acrylamide induces neurodegeneration that can be reversed by antioxidant plant essential oils, while the link between retinal degeneration and OS is supported by the protective role of cytochrome b5 overexpression, which reduces lipid peroxidation [82]. Among the mechanisms linking LP to neurodegeneration, 4-HNE was identified as a trigger and its levels in an animal model increased following induction of neurodegenerative lesions [82].

In the animal model, both Parkinson’s disease and Friedreich’s ataxia were associated with dysregulated mitochondrial metabolism and increased lipid peroxidation, and they could be ameliorated with antioxidants. In Huntington’s disease (HD), an increase in 4-HNE levels in different brain areas was also observed [82].

Alzheimer’s disease (AD) is certainly the most investigated in connection with LP. In human studies, excessive ROS generation was reported under AD conditions, and it was found to be associated with β-amyloid aggregation, while, in turn, β-amyloid plaques produce ROS and LP, and increased OS biomarkers are found in association with AD [84]. Histological analysis revealed co-localization of LP secondary products and β-amyloid plaques in the brain, while there is a relationship with oxidized low-density lipoprotein (LDL) levels in AD [84]. Owing to this strong association, LP markers can be used both as diagnostic tools for AD and to monitor the efficacy of AD treatments [84]. Formation of Schiff bases of the cholesterol oxidation product 3-β-hydroxy-5-oxo-5,6-secocholestan-6-al and β-amyloid is known to be amyloidogenic and it was found that this modification, occurring in specific sites, has a different influence on aggregation kinetics, particularly adducts at Lys-16, obtained at physiological levels of β-amyloid, enabling both kinetic and thermodynamic aggregation that are sufficient to form neurotoxic lesions [65]. Based on the above, antioxidants are among the most promising therapeutic approaches for AD. Among them, metal chelators (acting as preventive antioxidants, vide infra), indirect antioxidants acting through the activation of the Nrf2 transcription factor, which induce de novo synthesis of antioxidant enzymes, and plant-based antioxidants like curcumin, resveratrol, capsaicin, epigallocatechin gallate (EGCG) from green tea and quercetin have been more extensively investigated, with favorable clinical outcomes [15]. Naringenin, a flavanone obtained mainly from grapefruit (*Citrus paradisi*) and other citrus fruits, possesses neuroprotective activity, along with anti-inflammatory effects, and it has beneficial effects on learning and memory in the AD model, through the mitigation of lipid peroxidation [15].

### 4.5. LP and Ferroptosis

Ferroptosis, first identified by Stockwell in 2012 [8], is the last of several forms of programmed cell death, differing from apoptosis, which have been described in recent decades. It owes its name to the initial attribution as iron-dependent oxidative cell death, promoted by a void in the antioxidant defenses based on Cys, which could be artificially induced by erastin, an inhibitor of cystine uptake working by irreversible blocking of the cystine/glutamate antiporter system (x_c_^−^), and it could be prevented by ferrostatin-1 [8], an iron-chelating aromatic amine that is instead unable to inhibit apoptosis. The discovery that, in mice, the knockout of selenoenzyme glutathione peroxidase 4 (GPX4)—a specific phospholipid hydroperoxide glutathione peroxidase that differs from other GPXs preferably reducing hydrogen peroxide—would cause cell death by inducing massive ferroptosis, which would be inhibited by liproxstatin-1, allowed recognizing the key role of GPX4 as a controller of ferroptosis [14,85]. The role of iron in ferroptosis appears linked to its ability to initiate LP via the Fenton reaction (Figure 1), by decomposing phospholipid hydroperoxides, which in turn could be the product of LP or formed enzymatically, e.g., by lipoxygenase (LOX) [11]. Although it was initially identified as a key player in the definition of ferroptosis, the actual necessity or dominance of iron in control of ferroptosis remains unclear [86]. Instead, the subsequent recognition that ferrostatin-1 and lipoxtatin-1 would act essentially as chain-breaking antioxidants (vide infra) in the inhibition of ferroptosis [12], and that other potent antioxidants like phenoxazines, diarylamines and nitroxides would also inhibit ferroptosis, points toward the prominent role of LP in ferroptosis, besides it being initiated by iron or by other processes [11,12,86]. Other antioxidants such as vitamin E [86] and vitamin K [87] were also found to inhibit ferroptosis. In other words, it is LP that drives the cell death [11] and the many degenerative diseases that are linked to ferroptosis. Regulation of LOX biosynthesis and of the LOX-mediated accumulation of hydroperoxides was identified as a key point in the control of ferroptosis [80]; however, its actual significance has lately been challenged by showing that, in different LOX-overexpressing cells, only some well-known LOX inhibitors were able to counteract ferroptosis and, incidentally, they were all effective chain-breaking antioxidants, likely acting through inhibition of LP [88]. Ferroptosis is related to cancer, which can be modulated by ferroptosis inducers or inhibitors [89]. Ferroptosis is also associated with neurodegenerative conditions such as AD, Parkinson’s disease, Friedreich’s ataxia and Huntington’s disease [90], cardiovascular diseases and diseases of the urinary system [91]. Therefore, modulating ferroptosis in both directions, either by triggering it or by inhibiting it with antioxidants, might offer a potent tool in the therapy of many such diseases [90,91].

## 5. Antioxidants

Antioxidants are a very heterogeneous class of compounds, small molecules and enzymes that share the task of protecting oxidizable molecules or materials from oxidative transformation. In the biological context, the reference oxidative process is lipid peroxidation (LP); therefore, antioxidants are typically defined and discussed on the basis of their ability to prevent, slow down or block LP [3,21]. Based on where and how they interfere with the LP radical chain, antioxidants are classified as *preventive*, if they impair the initiation process, and *chain-breaking* if they block or slow down the propagation, while a new category, the *termination-enhancing* antioxidants, was recently introduced by our group, to include those molecules, such as some terpenes and terpenoids from essential oils, which act by favoring the radical-chain termination without actually impairing propagation [21,92,93].

### 5.1. Preventive Antioxidants

Antioxidants in this class are extremely heterogenous, ranging from small molecules to complex enzyme systems. They prevent the onsetting of LP by preventing the formation of the initial alkyl radical, which would give rise to the propagation cycle. Since triggering events can be different, their prevention occurs by a multitude of mechanisms, each addressing one specific mode of radical initiation. For instance, UV filters (sunscreens) might protect the skin by photoinitiated LP (i.e., preventing the action of photosensitizers) [21], but they would not affect radical initiation due to metal-catalyzed decomposition of peroxides and hydroperoxides (e.g., via the Fenton reaction, see Section 2.1). The latter is a prevailing mechanism of initiation, which explains the evolutionary development of enzyme systems like peroxidases aimed at clearing the biological medium of peroxides and hydroperoxides. Among them, catalase (CAT), which catalyzes the dismutation of H_2_O_2_ into water and molecular oxygen, and thiol peroxidases like GPX, which reduce hydroperoxides and hydrogen peroxide to alcohols or water, respectively, using a cysteine-derived thiol (e.g., the tripeptide glutathione) as the sacrificial reducing agent, can be classified as preventive antioxidants, since they “fuel down” the initiation process [21]. Along this line, superoxide dismutases (SODs), clearing superoxide radicals, and glutathione reductase (GR), which reduces oxidized glutathione using NADPH as the reducing agent, can also be assigned to the same class [21].

Metal chelators are also important members of this community. They block transition metal ions like iron and copper in a less redox-active form, allowing their indispensable presence in biological systems, yet neutralizing their ability to undergo Fenton-like chemistry. They can do so in case the two states participating in the redox cycle (e.g., Fe^2+^ and Fe^3+^) form with the chelator complexes with sufficiently different stability. For instance, most chelators have higher affinity for Fe^3+^ than for Fe^2+^, therefore chelated Fe^3+^ would have a reduction potential diminished by the difference in formation free energy (∆∆*G*_form_ = ∆*G*_form_^3+^ − ∆*G*_form_^2+^) between Fe^3+^ and Fe^2+^ complexes, which would make the redox cycle thermodynamically not viable, blocking iron in the oxidized form. Examples of chelating agents of biological relevance are transferrin and ceruloplasmin [21].

### 5.2. Chain-Breaking Antioxidants

Chain-breaking antioxidants, also named radical trapping antioxidants (RTAs), are certainly the biggest class of small-molecule antioxidants in all fields of application, and *phenols* are the prototypical members [94,95]. In cells, α-tocopherol (α-TOH) is certainly the most important representative and the most potent lipophilic antioxidant, often used as the benchmark in antioxidant research [3]. The identification of the mechanism of its antioxidant action and of the relative reactivity of different tocopherols (α, β, γ, δ) highlighting the parallelism between vitamin E activity in vivo and the relative antioxidant activity [3], allowed understanding of the structure–activity relationship governing its properties and linked vitamin E to the knowledge developed with simpler synthetic phenolic antioxidants [3,21,94,95,96,97].

RTAs act by trapping alkylperoxyl radicals, thereby competing with chain propagation. In order to be effective, the reaction of RTAs with ROO• must be (much) faster than the rate of chain propagation, i.e., the rate at which peroxyl radicals attack the lipids (Equation (3)). Therefore, chain-breaking antioxidant activity works on purely kinetic bases, being a competition between two reactions. The rate constant of inhibition *k*_inh_ for trapping ROO• by the antioxidant (Equation (3)) is the key parameter deciding the effectiveness of an antioxidant and, since the antioxidant has normally much lower concentration that the oxidizable substrate it is called to protect (e.g., membrane lipids), *k*_inh_ >> *k*_p_ is necessary. For instance, to protect linoleic acid residues (*k*_p_ = 62 M^−1^s^−1^, see Table 1) with 1% antioxidant relative to lipids, *k*_inh_ ≥ 10^4^ M^−1^s^−1^ is required [21].

(3) $$V_{trapping}>V_{propagation}\overset{}{\Rightarrow }\;k_{inh}\left[Antioxidant\right]>k_{p}[Substrate]$$

**Figure 9:**
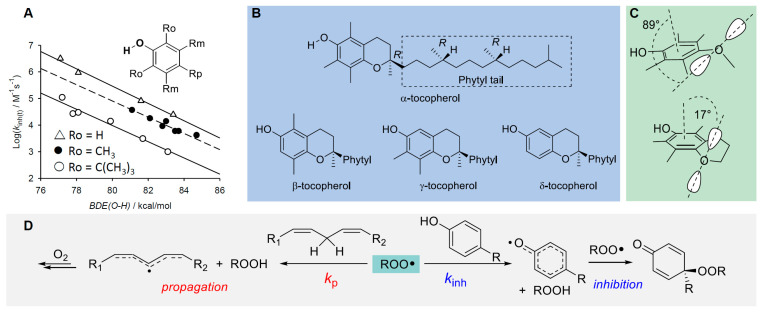
Linear free energy correlations between rate constant for ROO• trapping and BDE_OH_ for phenolic antioxidants bearing different ring substituents (**A**); structure of the main tocopherol congeners forming vitamin E (**B**); role of stereoelectronics in maximizing the ED contribution of -O- substituent included in the chroman ring or free to rotate (**C**); and representation of the kinetic competition involving chain-breaking antioxidants (**D**).

It should be noted that only the trapping of ROO• is relevant to afford antioxidant protection, as they are the sole chain-propagating species (with the exception of HOO•, see Section 2.2). In the case of phenols like α-TOH, the reaction with ROO• occurs by formal HAT from the phenolic -OH group, to afford a stabilized phenoxyl radical α-TO•, which is normally unable to propagate the radical chain. Instead, it is sufficiently long-lived to “wait” in solution to trap a second ROO•, normally by addition to the aromatic ring, as illustrated in Figure 9. Therefore, one molecule of (mono)phenolic antioxidant can break two radical chains, or it has a *stoichiometric factor n* = 2. This is the second most important parameter in quantifying antioxidant performance.

The rate of peroxidation during full inhibition by an antioxidant AH is given by Equation (4), where *R*_i_ is the rate of radical initiation.

(4) $$-\frac{d\left[O_{2}\right]}{dt}=\frac{d\left[ROOH\right]}{dt}=\frac{k_{p}\left[RH\right]R_{i}}{nk_{inh}\left[AH\right]}+R_{i}$$

The rate constant *k*_inh_ depends on the BDE of the phenolic O-H being broken and of the ROO-H bond of newly formed hydroperoxide, which is worth about 88 kcal/mol [21,94]. Hence, phenols with lower BDE would give a more exothermic and faster reaction (BDE is 77.1 kcal/mol for α-TOH [94]). There exist linear free energy correlations between the BDE and *k*_inh_ for phenolic antioxidants, which also account for steric hindrance in *ortho* to the reactive -OH, as depicted in Figure 9 [21,94,95]. Substituents in the phenolic ring determine the reactivity with peroxyl radicals, according to their electronic properties: electron-donating groups (EDGs) decrease the bond dissociation enthalpy (BDE) of the phenolic O-H, making the HAT to peroxyl radicals faster, while electron-withdrawing groups (EWGs) have the opposite effects [21,94]. The effect is more marked when the substituent is in the *ortho* or *para* position, conjugated with the -OH group, while it is less important in the *meta* position and, in a first approximation, the effect of each substituent in decreasing the BDE is additive [94]. Stereoelectronic effects are also important; indeed, α-TOH has lower BDE and much higher *k*_inh_ than equivalently substituted phenols, due to the almost parallel alignment of one lone pair of the RO- substituent with respect to the axis of the aromatic π-system, forced by the geometric constraints in the chroman structure (Figure 9). The performance of synthetic or natural monophenolic antioxidants, including currently popular compounds like bakuchiol [95] and curcumin derivatives [37], can be rationalized with the above concepts. The values of BDE, *k*_inh_ and *n* for some representative antioxidants are listed in Table 2.

#### 5.2.1. Insertion of N(s) in the Phenolic Ring: 3-Pyridinols and 5-Pyrimidinols

**Figure 10:**
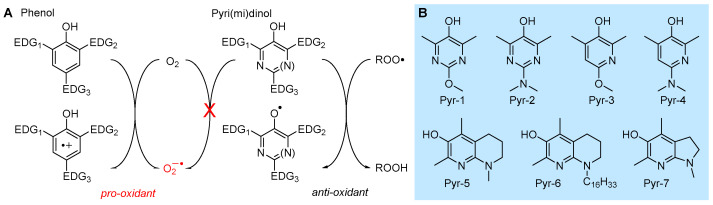
Different reactivity with oxygen and ROO• of electron-rich phenols and pyri(mi)dinols (**A**) and examples of 3-pyridinol and 5-pyrimidinol antioxidants (**B**).

Research aimed at the rational design of phenolic antioxidants that would be more effective than α-TOH, using the strategies outlined above, e.g., by inserting stronger ED substituents like the amino group and forcing its conjugation by geometrical constraints, eventually showed its limits. In parallel to decreasing the BDE and hence increasing the reactivity with ROO•, ED substituents also decrease the ionization potential (IP) of the molecule, to the point it directly reacts with molecular oxygen by electron transfer (ET) to yield the superoxide radical (O_2_^−•^) (Figure 10). Therefore, these compounds are pro-oxidant and toxic, besides being terribly unstable under normal handling conditions [98]. In a joint effort with Pratt’s group, we found that replacing one or two >CH moieties in the aromatic ring with nitrogens in non-conjugated positions (i.e., 3 and 5), to afford the corresponding 3-pyridinols and 5-pyrimidinols, would increase the BDE_OH_ by about 1.1 kcal/mole per nitrogen (∆BDE of +1.1 kcal/mol and +2.5 kcal/mol for 3-pyridinols and 5-pyrimidinols, respectively), while the IP would increase 10-fold more (∆IP of +11 kcal/mol and +24 kcal/mol for 5-pyrimidinol) [98,99]. In other words, insertion of heteroaromatic nitrogen(s) expands the thermodynamic gap between the two competing reaction pathways, HAT to ROO• and ET to O_2_, while maintaining the same substituent effects known for phenols [99]. Two features favored this strategy in antioxidant design: (1) the slightly higher BDE_OH_ that would disfavor the reactivity can be compensated with stronger ED substituents, like the amino group (inaccessible in the phenolic series), without compromising the stability toward oxygen [99]; (2) 3-pyridinols and 5-pyrimidinols are more reactive toward ROO• than phenols having an identical BDE, owing to the intervention of polar effects stabilizing the transition state [100]. This led to the discovery of a wealth of potent RTAs (some examples in Table 2), many of which easily outperformed α-TOH despite the much simpler structure and easier synthetic accessibility [98,99,100,101,102,103,104,105,106], which also included the most potent chain-breaking antioxidant ever reported, able to quench peroxyl radicals at a diffusion-controlled rate (Pyr-7 in Figure 10) [101].

#### 5.2.2. Solvent and Medium Effects in Chain-Breaking Antioxidant Activity

**Figure 11:**
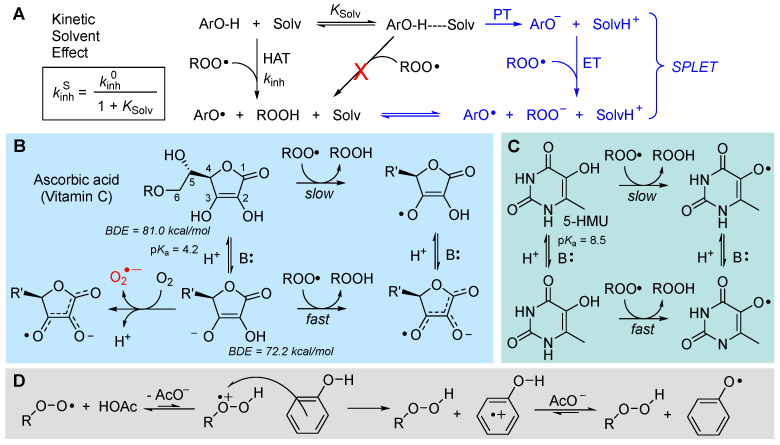
Solvent effects on the reactivity of antioxidants, showing the possible mechanistic change in protic solvents (**A**), pH-dependent antioxidant chemistry of ascorbic acid (**B**) and of 5-hydroxymethyluracil (**C**) and acid catalysis in the antioxidant activity of phenols in polar solvents (**D**).

The rate of radical trapping by phenols and most other RTAs is subject to solvent effects [107], which must be carefully considered in testing antioxidant activity [108] or in planning antioxidant strategies. In hydrogen-bond-accepting (HBA) solvents, *k*_inh_ is apparently decreased by as much as two orders of magnitude, depending on the actual HBA ability of the solvent (not its polarity) [107]. The effect does not depend on the radical reacting with the antioxidant (e.g., R•, RO•, ROO•) but on the ability of the antioxidant to act as a hydrogen-bond donor (HBD) to the solvent [109], since the effect is due to formation of an antioxidant–solvent complex in which the “active” -OH group (or, in general, the -XH group transferring the H-atom to radicals) is “blocked” by H-bonding to the solvent, and only the fraction of non-H-bonded antioxidant at equilibrium is available to react with radicals (Figure 11) [106,107]. Different antioxidants will have different sensitivity to solvent effects.

The HBD ability of the antioxidant and the HBA ability of the solvent can be quantified by Abraham’s solvatochromic parameters α_2_^H^ and β_2_^H^, respectively [110,111], and solvent effects can be accounted for quantitatively by the Ingold–Snelgrove equation (Equation (5)), which allows predicting *k*_inh_ in any solvent (*k*_inh_^S^) once it is known in a non-H-bonding solvent (*k*_inh_^0^) [112].

(5) $$Log(k_{inh}^{S}/M^{-1}s^{-1})=Log(k_{inh}^{0}/M^{-1}s^{-1})-8.3\times \alpha_{2}^{H}\beta_{2}^{H}$$

Triglycerides [24] and phospholipids [107] have strong HBA groups (i.e., the C=O and P=O), which visibly decrease the antioxidant protection by RTAs. In a heterogenous system of lipid particles in water, however, an additional factor affects the kinetics of antioxidant protection: the compartmentalization, which may render the rate of exchange of radicals and antioxidants among particles rate limiting. For instance, the value of *k*_inh_ for α-TOH measured in micelles of methyl linoleate and in phospholipid liposomes is, respectively, two orders of magnitude and three orders of magnitude lower than in homogenous non-H-bonding solution [21,107].

The decrease in the rate of formal HAT reaction to radicals in HBA solvents could favor other reaction mechanisms [113,114]. It has been shown that in protic solvents like alcohols, able to solvate anions, the reaction of acidic phenols (or other antioxidants) with radicals could take place via a mechanism named sequential proton loss electron transfer (SPLET), depicted in Figure 11, consisting in proton transfer (PT) to the solvent followed by ET from the electron-rich anion of the antioxidant to oxidizing radicals [113]. In water, this mechanism is favored in alkaline pH and can result in an increase in the rate of peroxyl radical trapping [114].

This change in the reaction mechanism does not occur only with phenols but controls the antioxidant activity of other biological antioxidants like 5-hydroxylmethyluracile (5-HMU) [115] and, most notably, of *ascorbic acid* and its derivatives [116,117], boosting their performance in the presence of a base (Figure 11). In the case of ascorbic acid, deprotonation also opens the way to its direct reaction with O_2_ to yield superoxide radicals, accounting for its instability in aqueous solution. This is also a pro-oxidant process that partly counteracts the antioxidant behavior; as a consequence, the overall antioxidant performance of ascorbic acid largely depends on the experimental settings, and it can be significantly improved by inclusion in inert nanocarriers [117].

Opposite to the base effect outlined above, phenolic (and pyridinolic/pyrimidinolic) antioxidants have been found to undergo acid catalysis in their antioxidant activity. The behavior is only observed in polar solvents and can largely boost the reactivity, depending on the phenol. It is due to a change in reaction mechanism involving the partial protonation of the ROO• radical to form a highly oxidizing species that takes one electron from the phenol and forms the antioxidant’s radical cation, which undergoes rapid acid–base exchange to afford the usual reaction products [118], as illustrated in Figure 11.

**Table 2:** Bond dissociation enthalpy (BDE), rate constants for ROO**•** radical trapping *k*_inh_ and stoichiometric factor *n* for representative antioxidants at 303 K in chlorobenzene.

Entry	Compound	BDE kcal/mol	*k*_inh_ M^−1^s^−1^	*n*	Ref.
1	α-Tocopherol	77.1	3.2 × 10^6^	2.0	[3,21]
2	β-Tocopherol	-	1.3 × 10^6^	2.0	[3,21]
3	γ-Tocopherol	-	1.4 × 10^6^	2.0	[3,21]
4	δ-Tocopherol	-	4.4 × 10^5^	2.0	[3,21]
5	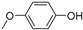	81.7	2.7 × 10^5^	2.0	[21,94]
6	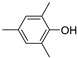	81.6	8.5 × 10^4^	2.0	[21,94]
7	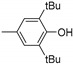	79.9	1.4 × 10^4^	2.0	[21,94]
8	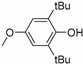	77.2	1.1 × 10^5^	2.0	[21,94]
9	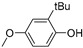	80.3	6.4 × 10^5^	1.8	[21,94]
10	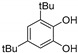	78.2	1.1 × 10^6^	2.0	[94,119]
11	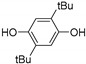	79.2	1.6 × 10^6 1^	0.3	[120]
12	Quercetin	-	5.5 × 10^5^	2.1	[119]
13	Pyr-1 ^2^	81.4	2.1 × 10^5^	2.0	[100]
14	Pyr-2 ^2^	77.1	8.6 × 10^6^	2.0	[100]
15	Pyr-3	78.9	4.4 × 10^5^	2.1	[106]
16	Pyr-4	75.9	1.6 × 10^7^	2.0	[101]
17	Pyr-5	75.2	8.8 × 10^7^	1.3–2.0	[101]
18	Pyr-6	75.2	8.8 × 10^7^	~2	[103]
19	Pyr-7	74.3	2.8 × 10^8^	~2	[101]
20	α-Selenotocopherol	78.1	1.2 × 10^6^	1.9	[121]
21	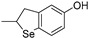	81,6	3.8 × 10^5^	2.0	[122]
22	Te-1	-	9.2 × 10^6^	0.4 ^6^	[123]
23	Te-2	78.9	1.0 × 10^7^	0.4 ^6^	[124]
24	Te-3	-	1.6 × 10^6^	0.3 ^6^	[125]
25	Te-4	-	1.0 × 10^7^	0.4 ^6^	[125]
26	Phenoxazine ^2,3^	76.1	2.9 × 10^7^	5	[94]
27	Phenothiazine ^2,4^	78.2	8.8 × 10^6^	1.8	[94]
28	Dia-1	78.8	3.4 × 10^7^	>2	[126]
29	Dia-2	79.0	3.7 × 10^7^	>2	[126]
30	Ferrostatin-1 ^5^	-	3.5 × 10^5^	2.0	[11]
31	Liproxstatin-1 ^5^	-	2.4 × 10^5^	1.9	[11]

^1^ *k*_inh_ is doubled by a statistical factor. ^2^ Data at 50 °C. ^3^ Values at 37 °C are: *k*_inh_ 4.1 × 10^7^ M^−1^s^−1^, *n* = 2.3 from ref. [11]. ^4^ Values at 37 °C are: *k*_inh_ 8.0 × 10^6^ M^−1^s^−1^, *n* = 2.1 from ref. [11]. ^5^ Data at 37 °C. ^6^ Without thiols.

#### 5.2.3. Polyphenols and Flavonoids

**Figure 12:**
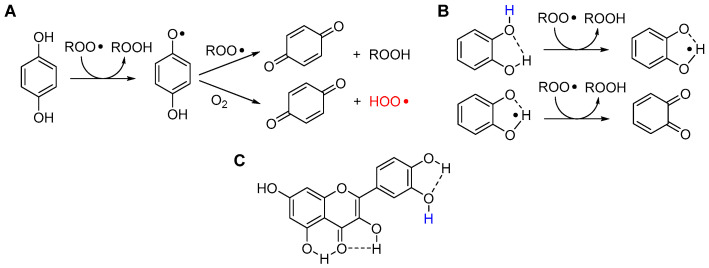
Radical reactivity of hydroquinone (**A**), of catechol (**B**) and structure of quercetin (**C**).

Hydroquinone (1,4-dihydroxybenzene) and catechol (1,2-dihydroxybenzene) are the two key structural motifs in polyphenols and flavonoids. Even the simple unsubstituted molecules have high reactivity with ROO• radicals due to the strong ED character of each -OH group toward the other in the conjugated position [21,120]. Both hydroquinones and catechols are nominally able to trap two ROO• radicals by stepwise transfer of the two O-H. However, their relative position (*ortho* or *para*) deeply affects the antioxidant behavior [120], as shown in Figure 12. In hydroquinones (e.g., ubiquinol or coenzyme QH_2_), the semiquinone radical formed upon transfer of the first H-atom has a very low BDE for the second O-H (~50 kcal/mol) and it is rather acidic (pKa ~ 4) [120]; therefore, via different mechanisms which have been discussed in detail [120] the semiquinone radical can react directly with O_2_ to form a hydroperoxyl radical (HOO•) or superoxide radical anion (O_2_^−•^) in lipidic medium or in water, respectively. This process, which parallels the generation of superoxide in the mitochondrial respiratory chain, has pro-oxidant action starting new oxidative chains; therefore, it partly counteracts (by shortening *n*) the otherwise very good antioxidant behavior of hydroquinones [120].

In catechols, the intramolecular H-bond makes one -OH unreactive, but it decreases the BDE of the unbound O-H, increasing its reactivity. The resulting semiquinone radical is stabilized by the intermolecular H-bonding, which prevents its reaction with O_2_. Overall, even the unsubstituted catechol traps two peroxyl radicals with *k*_inh_ similar to δ-TOH, being an excellent antioxidant [119,127]. Not surprisingly, the catechol moiety is highly conserved in natural flavonoids (Figure 12) and, in a lipidic environment, their antioxidant activity is largely dictated by the catechol ring. Indeed, despite the large number of phenolic -OH groups in their structure, the reactivity of most polyphenols is modulated by the occurrence of intramolecular H-bonds [128]. As a consequence, flavonoids can trap more than two ROO• radicals, but with different rate constants *k*_inh_, hence the trapping of the first two contributes most to the overall antioxidant performance [129]. In an aqueous environment, their reactivity is instead dictated by pH, and multiple mechanisms (PT-ET vs. HAT) can take place, as previously discussed in detail for quercetin [127].

#### 5.2.4. Synergy among Antioxidants and Tocopherol-Mediated Peroxidation (TMP)

In nature, as in the protection of human-made materials, antioxidants are never used alone and cooperative effects among antioxidants are often the key to successful protection. The most relevant example in cells is certainly the synergic interplay between vitamin E and vitamin C. α-TOH is confined in the lipid bilayers and protects phospholipids from ROO• attack, being oxidized to an α-TO• radical, while water-soluble ascorbate reacts with α-TO• at the lipid–water interface, reducing it back to the starting α-TOH and extending its protective duration [130]. On doing so, ascorbate is oxidized to the corresponding radical; therefore, besides the recycling of α-TOH, the importance of this process resides in the “exporting of the unpaired electron” outside the lipid membrane, out of the reach of highly oxidizable lipid components. Indeed, it has been demonstrated by Bowry and Ingold [131] that, in the autoxidation of human low-density lipoprotein (LDL), in the absence of ascorbate in the aqueous medium, the presence of α-TOH in the lipid core accelerates the peroxidation instead of blocking it—an apparently paradoxical behavior that they named tocopherol-mediated peroxidation (TMP)—due to the “excessive” resident time of α-TO• inside the lipid particle before it encounters a second ROO•, which gives time for the occurrence of even a slow and unfavored reaction of α-TO• with lipids, to (re)start the oxidative chain [131].

Interestingly, when pyridinol Pyr-6 was probed in place of α-TOH in the protection of LDL in the absence of vit C or co-antioxidants, it showed no sign of TMP, along with much higher antioxidant protection [103]. Cooperative effects similar to the vit E/vit C example also occur among phenolic and/or other antioxidants in homogenous solution, and their mechanism and efficiency have been thoroughly investigated [106,132].

#### 5.2.5. Phenols Bearing Organochalcogen Substituents

The insertion of chalcogens heavier than oxygen (S, Se and Te) as substituents in the *ortho* or *para* position in phenolic (and pyridinolic) structures is an important strategy to afford novel antioxidants with a distinctive reactivity toward oxidizing radicals [121,133]. It has highlighted how intramolecular H-bonding can be used to afford antioxidants whose reactivity can predictably be modulated by conformational constraints and by the solvent [119]. Notable examples are lipoic acid adducts of natural catechols like hydroxytyrosol or ditocopheryl sulfides and disulfides [121].

**Figure 13:**
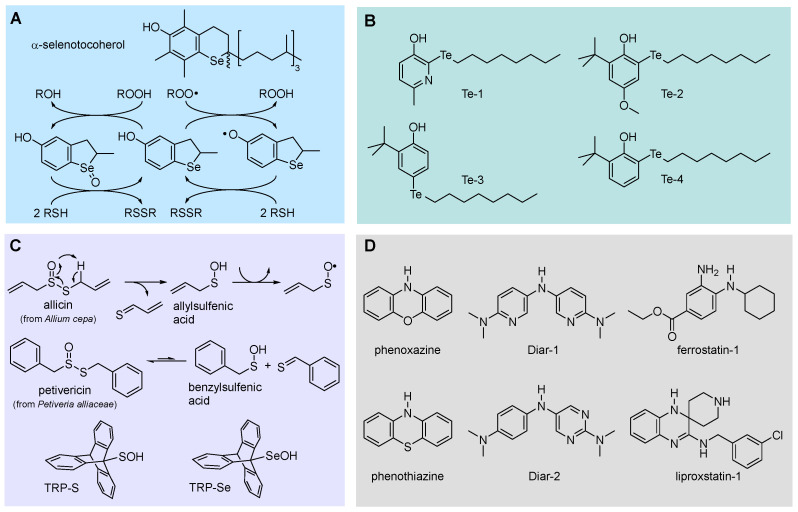
Examples of organochalcogen-containing phenolic antioxidants (**A**,**B**), sulfenic and selenenic acids (**C**) and diarylamine antioxidants (**D**).

Phenolic antioxidants cannot be recycled by thiols (unlike by ascorbate), which somewhat “wastes” the most abundant reducing source in biological systems. Looking for antioxidants that would boost this ability, we carried out a long cooperative project with Engman’s group focused on Se- and Te-substituted phenols. All-*rac*-α-selenotocopherol, in which the chromanol -O- was replaced by -Se-, disappointingly showed slightly higher BDE_OH_ and slightly lower *k*_inh_ than the natural counterpart, with no additional property toward thiols [121]. However, some of its simplified congeners like Se-1 (Figure 13), besides being potent chain-breaking antioxidants, could be recycled by *N*-acetylcysteine (NAC) at the water–lipid interface in a biphasic lipid peroxidation model system [122].

Insertion of alkyltelluro (RTe-) substituents in the *ortho* or *para* position in the phenol or in the 3-pyridinol structure affords even more interesting antioxidants (Figure 13). These compounds can be recycled by thiols like cysteine derivatives both in homogenous lipid solution and heterogenous (aqueous biphasic) systems [123,124,125]. Additionally, even simple structures have a *k*_inh_ for trapping ROO• radicals much higher than reference α-TOH and their reactivity does not respond to the correlation with the BDE_OH_ reported in Figure 9 or to the solvent effect described in Figure 11. This proves a different reaction mechanism involving an oxygen-atom transfer from ROO• to RTe-, for which we refer to the original literature [125]. Interestingly, the efficiency of regeneration by thiols is much lower than 1, meaning that the same compounds could also be used as pro-oxidant agents in biological systems, acting through the depletion of Cys-derived thiols like glutathione [125]. This is particularly intriguing in the light of the role that this mechanism has in triggering ferroptosis [8].

#### 5.2.6. Sulfenic and Selenenic Acids

*Allium* plants have long been regarded as a rich source of antioxidants, particularly owing to the distinctive content of sulfurated volatile components. Among them, thiosufinates like allicin and petivericin (Figure 13) have been demonstrated to be potent antioxidants [134,135]. This is due to the release of unstable sulfenic acids (RSOH), which would rapidly trap ROO• radicals to afford stabilized sulfenyl radicals (RSO•). Owing to their instability that makes their isolation prohibitive, the reactivity of sulfenic acids has remained largely unknown, despite their major relevance in biological systems, since they are involved in cellular redox homeostasis, being formed upon oxidation (e.g., by H_2_O_2_) and fragmentation of cystine. Only recently did the synthesis of stable tripticenesulfenic acid (TRP-S, Figure 13) shed light on their antioxidant chemistry [134]. With a BDE_OH_ as low as 71.9 kcal/mol, they trap ROO• radicals with *k*_inh_ in the range of 3 × 10^6^ M^−1^s^−1^ from sterically hindered TRP-S to 3 × 10^7^ M^−1^s^−1^ for benzylsulfenic acid from petivericin and up to ~1 × 10^8^ M^−1^s^−1^ for allylsulfenic acid from allicin, i.e., they are among the most potent antioxidants in nature [135].

Their heavier homologues, the selenenic acids (RSeOH), are involved in the redox cycle of the GPX enzyme; however, they are even less stable than sulfenic acids. Synthesis of hindered tripticeneselenenic acid (TRP-Se, Figure 13) allowed clarifying their properties and reactivity, showing a higher BDE_OH_ (80.9 kcal/mol) compared to sulfenic acids, yet having unexpectedly high reactivity toward ROO•, i.e., *k*_inh_ = 1.7 × 10^5^ M^−1^s^−1^ for sterically hindered TRP-Se (in PhCl at 30 °C) [136]. This is 18-fold lower than that of TRP-S despite the 9 kcal/mol higher BDE [136]. Unfortunately, the instability of these compounds hampers their use as antioxidants, unless they can be generated in situ from suitable precursors.

#### 5.2.7. Aromatic Amines and Diarylamines as RTAs

Aromatic amines such as diarylamines are, after phenols, the main class of chain-breaking antioxidants. Owing to the higher thermal stability and lower reactivity compared to phenols, the most common substituted diphenylamines have mainly been used as antioxidants in high-temperature processes. However, their tricyclic analogues, such as phenoxazine and phenothiazine, have instead very high antioxidant activity at close to ambient temperature, owing to better conjugative stabilization of the aminyl radical formed upon quenching ROO• [21]. Insertion of heterocyclic nitrogens in the structure of diphenylamine, i.e., extending the same design strategy previously described for phenols (see Section 5.2.1), has created a large family of outstanding antioxidants, containing up to four heterocyclic nitrogens per molecule and a wealth of ED substituents, with large structural variability [98,126,137,138]. Many such molecules largely outperform reference α-TOH in terms of *k*_inh_, in some cases having very low to negligible activation energy for ROO• trapping [126], implying negligible temperature dependence of the antioxidant activity [98]. Perhaps most notably, they outperform α-TOH in terms of the number of trapped ROO• radicals, having large stoichiometric factors that depend on temperature, due to a regeneration cycle involving the transient formation of the corresponding nitroxyl radicals [49].

Interestingly, recent work from Pratt’s group has demonstrated that several antioxidants belonging to this class, namely diarylamines, phenoxazines and phenothiazines, including those displayed in Figure 13, are potent inhibitors of ferroptosis, able to match or surpass the performance of reference inhibitors liproxstatin-1 and ferrostatin-1 (Figure 13), which incidentally also belong to the class of aromatic amines [11].

#### 5.2.8. Unconventional Antioxidant Mechanisms and HOO• as Co-Antioxidant

**Figure 14:**
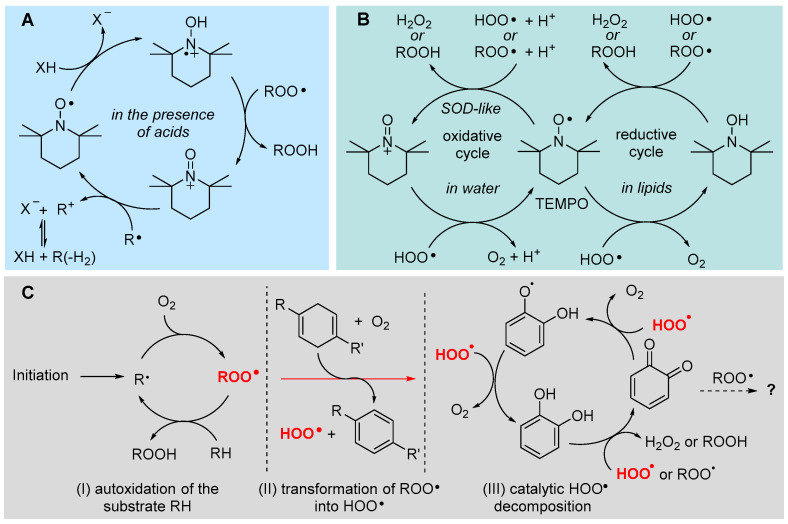
Non-conventional antioxidant mechanisms involving persistent nitroxides in acidic medium (**A**), or in the presence of HOO• (**B**), or involving *ortho*-quinones in the presence of HOO• (**C**).

Sterically hindered dialkyl nitroxides like the 2,2,6,6-tetramethylpiperidin-1-oxyl radical (TEMPO) are sufficiently persistent to be manipulated like a “normal” molecule, which has allowed an enormous body of investigation. Among the many applications, a wealth of studies have shown a beneficial activity in a multitude of pathological conditions commonly associated with oxidative stress, which has suggested an antioxidant activity that has long escaped rationalization [139,140]. In water, TEMPO was demonstrated to behave as a SOD mimic, being able to decompose superoxide and peroxyl radicals via a catalytic cycle with the intermediation of the oxoammonium ion [141,142] (Figure 14). However, early knowledge indicated that nitroxides are unable to trap peroxyl radicals in a lipidic environment and have negligible antioxidant activity toward lipids.

We were surprised to find that they can indeed be excellent antioxidants in lipidic or organic media, but only in the presence of acids [143]. Of interest, depending on the strength of the acid, they could behave as stoichiometric antioxidants (trapping one radical per nitroxide) or they could be regenerated and work in a catalytic fashion if the acid is weak and forms a nucleophilic conjugated base, like acetic acid [144]. This chemistry, summarized in Figure 14, clearly helps explain their redox-related bioactivity, as carboxylic acids are abundant in biological systems, e.g., in proteins [144]. Even more surprising was the recent discovery of another catalytic antioxidant cycle of nitroxides, which is only expressed in lipidic or apolar environments, although it apparently resembles the SOD mimic behavior expressed only in water. The overall catalytic cycle occurs only in the presence of HOO• radicals (the neutral form of superoxide) and it does not involve the intermediacy of the oxoammonium ion from the nitroxide [145]. It was clarified, independently by our group and Pratt’s group, that the cycle occurs by *reduction* of the nitroxide (>NO•) to the parent hydroxylamine (>NOH), which is then reoxidized to the nitroxide by HOO• or by ROO• [49,145] (Figure 14). Therefore, HOO•, normally regarded as an oxidizing chain-carrying radical, here works as sacrificial *reducing* agent or as a *co-antioxidant*. It can be formed spontaneously during the peroxidation of lipids (see Section 2.4.3), or a dedicated source like cyclohexadiene or γ-terpinene can be added along with the nitroxide, to form a co-antioxidant system [145]. Interestingly, this co-antioxidant system is among the most potent ever reported, largely outperforming α-TOH [145]. This chemistry is likely to have major relevance in the control of ferroptosis and some preliminary success in this direction has recently been reported [12].

Unfortunately, no nitroxide is currently approved for medicinal use; however, we were intrigued to find that *ortho* quinones, the oxidized “waste” of catechol-type polyphenolic antioxidants, can undergo a similar chemistry (Figure 14). In the presence of spontaneously generated HOO•, or of an added source, *ortho*-quinones (*o*-Q) (more efficiently than *para*) are stepwise reduced to the semiquinone radical (*o*-QH•) then to the catechol (*o*-QH2), which can then perform its antioxidant action trapping both HOO• and ROO•. Albeit not as efficient as that involving nitroxides, the synergic combination of *o*-Q/γ-terpinene or *o*-QH2/γ-terpinene has shown superior protection of highly oxidizable polyunsaturated lipids [146]. The kinetic aspects regulating this chemistry have been clarified and found to be key to the purported antioxidant behavior of melanin biopolymers [47]. Possibly, this chemistry would show its potential in the control of ferroptosis, an issue currently under investigation in our group.

### 5.3. Termination-Enhancing Antioxidants

Some hydrocarbons, aldehydes and other simple highly oxidizable molecules, such as many terpenic components of plant essential oils (e.g., γ-terpinene, limonene, linalool and citral), when subjected to some radical source, undergo autoxidation that is characterized by very fast chain termination [147]. As a consequence, if they are mixed with other highly oxidizable substrates, they will promptly co-oxidize but increase the overall rate of chain termination, thereby reducing the overall rate of autoxidation and slowing down the degradation of the oxidable substrate [147]. These compounds are unable to block the chain propagation, nonetheless they have antioxidant behavior, which we classified as termination-enhancing [147]. This mechanism, which is typical of many non-phenolic essential oil components is, however, less effective than the chain-breaking activity possessed, for instance, by phenolic essential oil components [148] and it largely depends on the experimental conditions. It has non-monotonic dependence on the concentration of the antioxidant and it can become pro-oxidant at a high concentration [147].

### 5.4. Indirect Antioxidants

This class is often used to group those mechanisms that cannot take place within a material or in a test tube but require a living cell or organism. Indeed, many molecules which might show antioxidant behavior in cells, e.g., by reducing the markers of lipid peroxidation or the related biological damage, do not trap peroxyl radicals at a significant rate and would be unable to directly protect lipids from peroxidation; however, they are able to enhance the antioxidant defenses in a living organism, e.g., by inducing the biosynthesis of antioxidant enzymes. Typical examples are isothiocyanates from brassica vegetables [149,150], but the list can be extended to many plant-derived compounds, such as essential oil components [150,151]. Indeed, even flavonoids like quercetin, having good direct (chain-breaking) antioxidant activity, can also boost the antioxidant enzymes in cells [152], therefore direct and indirect activity can co-exist and have different weight depending on the experimental settings. One main mechanism by which indirect activity is accomplished is via the Nrf2 signaling system [150], which has been illustrated in Section 4.2.

## 6. Conclusive Remarks and Future Perspective

Research in the broad area of lipid peroxidation and antioxidant protection has taken place during the past 70 years, with variable emphasis. In the biomedical area, the disappointing outcomes of early clinical studies aimed at using antioxidants as drugs in the treatment of those degenerative conditions lacking other curative pharmacological solutions had somewhat, gradually, curbed the enthusiasm. Lack of success might have been due to a combination of factors, such as incomplete understanding of the role of oxidative stress and lipid peroxidation in physiology and disease, incomplete understanding of the double-sided antioxidant–pro-oxidant behavior of some molecules in biological systems, a misjudgment in the strategy of using an antioxidant when a pro-oxidant would have been the more effective choice and, last but not least, the choosing of antioxidants that were not fit for purpose. The recognition of ferroptosis as a new LP-based form of programmed cell death has revived the area and enormously boosted the search for molecules able to address OS-related pathologies via the modulation of ferroptosis. Now, understanding of the interplay between LP and pathophysiology is more mature and a wealth of extremely potent new antioxidants have become available. Pyridinols, diarylamines and heavy organochalcogen-substituted phenols have underexplored potential in this regard. Additionally, non-conventional antioxidant systems based on the exploitation of the endogenous HOO**•**/O_2_^−**•**^ reducing system appear very promising in the modulation of ferroptosis, a research area we are currently set to address.

## Figures and Tables

**Table 1:** Rate constants for the peroxidation of lipids in bulk or in chlorobenzene at 303 K.

Lipid	*k*_p_/(2*k*_t_)^1/2^ 10^−5^ M^−1/2^s^−1/2^	*k*_p_ M^−1^s^−1^	2*k*_t_ 10^5^ M^−1^s^−1^	Ref.
Methyl stearate (18:0) ^1^	~0.8	~0.01	15	[21]
Methyl oleate (18:1)	89.0	0.89	10	[22]
Methyl linoleate (18:2)	2100	62.0	88	[22]
Methyl linolenate (18:3)	3900	236.0	360	[22]
Linoleic acid (18:2)	-	62	-	[23]
Arachidonic acid (20:4)	-	197	-	[23]
Eicosapentaenoic ac. (20:5)	-	249	-	[23]
Docosahexaenoic ac. (22:6)	-	334	-	[23]
Cholesterol	-	11	-	[23]
7-Dehydrocholesterol	-	2260	-	[23]
Squalene	2500	68.0	74.0	[24]
Sunflower oil (60% of 18:2)	3600	66.9	34.5	[24]
PLPC ^2^	-	16.6	1.27	[25]
DLPC ^3^	-	13.6 ^4^	1.02	[25]

^1^ Data for hexadecane. ^2^ 1-Palmitoyl-2-linoleoyl-*sn*-glycero-3-phosphocholine; data at 310 K. ^3^ 1,2-Dilinoleoyl-*sn*-glycero-3-phosphocholine; data at 310 K. ^4^ For intermolecular propagation, *k*_p_ (intramolecular) = 5.1 s^−1^.
